# Bat-transmitted Human Rabies Outbreaks, Brazilian Amazon

**DOI:** 10.3201/1208.050929

**Published:** 2006-08

**Authors:** Elizabeth S.T. da Rosa, Ivanete Kotait, Taciana F.S. Barbosa, Maria L. Carrieri, Paulo E. Brandão, Amiraldo S. Pinheiro, Alberto L. Begot, Marcelo Y. Wada, Rosely C. de Oliveira, Edmundo C. Grisard, Márcia Ferreira, Reynaldo J. da Silva Lima, Lúcia Montebello, Daniele B.A. Medeiros, Rita C.M. Sousa, Gilberta Bensabath, Eduardo H. Carmo, Pedro F.C. Vasconcelos

**Affiliations:** * Instituto Evandro Chagas, Belém, Brazil;; † Instituto Pasteur, São Paulo, Brazil;; ‡ Secretaria de Saúde do Estado do Pará, Belém, Brazil;; § Ministério da Saúde, Brasília, Brazil;; ¶ Universidade Federal de Santa Catarina, Florianópolis, Brazil;; # Universidade Federal do Pará, Belém, Brazil

**Keywords:** human rabies virus, bat transmission, antigenic and genetic characterization, Brazilian Amazon

## Abstract

We describe 2 bat-transmitted outbreaks in remote, rural areas of Portel and Viseu Municipalities, Pará State, northern Brazil. Central nervous system specimens were taken after patients' deaths and underwent immunofluorescent assay and histopathologic examination for rabies antigens; also, specimens were injected intracerebrally into suckling mice in an attempt to isolate the virus. Strains obtained were antigenically and genetically characterized. Twenty-one persons died due to paralytic rabies in the 2 municipalities. Ten rabies virus strains were isolated from human specimens; 2 other cases were diagnosed by histopathologic examination. Isolates were antigenically characterized as *Desmodus rotundus* variant 3 (AgV3). DNA sequencing of 6 strains showed that they were genetically close to *D. rotundus*–related strains isolated in Brazil. The genetic results were similar to those obtained by using monoclonal antibodies and support the conclusion that the isolates studied belong to the same rabies cycle, the virus variants found in the vampire bat *D. rotundu*s.

Rabies virus, the prototype species in the family *Rhabdoviridae*, is a single-stranded, RNA, negative-sense, nonsegmented virus of the genus *Lyssavirus*; it is the causal agent of rabies, a disease that has been poorly studied in most developing countries (*1**,**2*). Nonetheless, Brazil has implemented rabies control measures, and urban human rabies, transmitted by dogs and cats, has decreased from 73 cases in 1990 to 17 cases in 2003 (*3*). Carnivores and bats are the primary reservoirs of rabies virus in all continents, and bat-transmitted rabies is relatively commonly diagnosed in Latin American and Caribbean countries (*1**,**4**–**6*). In Brazil, a small number of human cases have been confirmed as having been transmitted by vampire bat bites (*7**,**8*), but because surveillance has improved in the last few years, the occurrence of sporadic episodes suggests a situation similar to that observed in other American countries (*5**,**9**,**10*). Indeed, ≈39 cases of human rabies have been reported in the United States since the 1950s, and cases of rabies transmitted by vampire bat bites are commonly reported in Mexico, Chile, Colombia, Peru, Venezuela, and other New World countries (*1**,**5**,**6**,**9**,**11**,**12*).

Although rabies control measures have improved in many South American countries, the transmission of the disease by bats has increased and has become a public health concern, and several human cases have been detected. Outbreaks of bat-transmitted rabies have occurred in several remote areas in Peru (*5**,**13*), Venezuela (*9*), and more recently in Brazil (*7*). This article reports the results of an epidemiologic investigation and the antigenic and genetic characterization of rabies virus isolated during outbreaks of the bat-transmitted disease that occurred in March and May 2004 in remote areas of Portel and Viseu municipalities, respectively, in Pará State, Brazilian Amazon region.

## Materials and Methods

### Patients

**Figure 1:**
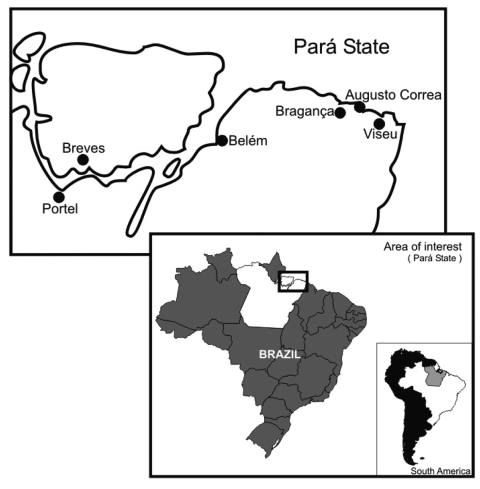
Pará State, showing the municipalities of Portel and Viseu, where vampire bat–transmitted rabies cases were reported. Source: Adapted from Government of Pará State website, http://www.brasilrepublica.hpg.ig.com.br/para.htm

All patients reported vampire bat bites several weeks or months before manifesting encephalitic symptoms. All were poor persons who lived in primitive conditions in the Acuty-Perera River communities in Portel or in the Curupati community in the municipality of Viseu, both in Pará State, Brazilian Amazon region (Figure 1). All patients exhibited a similar disease pattern, characterized by acute ascendant paralytic encephalitis. Twelve patients were hospitalized and after their deaths, an autopsy was performed and central nervous system (CNS) specimens were obtained for laboratory diagnostic procedures. Patient ages ranged from 2 to 58 years. Detailed information concerning the patients is shown in the Table.

**Table:** Generic information regarding 12 patients with diagnosis of paralytic rabies from Portel and Viseu municipalities, Pará State

Patient	Age (y), sex	Place	Municipality	Virus isolation	Onset	Death	Place of bite(s)	GenBank accession no.*
1	11, F	Tauaçu	Portel	Yes	19 Mar	28 Mar	Feet	NA
2	22, F	Ajará	Portel	Yes	9 Mar	24 Mar	Feet	DQ097077
3	42, M	Ajará	Portel	Yes	10 Mar	31 Mar	Feet	NA
4	30, M	Ajará	Portel	Yes	19 Mar	30 Mar	Feet	NA
5	12, M	Ajará	Portel	Yes	9 mar	30 Mar	Feet	DQ097076
6	26, F	Ajará	Portel	Yes	19 Mar	1 Apr	Feet	DQ097080
7	30, F	Laranjal	Portel	Yes	23 Mar	3 Apr	Feet	DQ097078
8	2, M	Laranjal	Portel	Yes	1 Apr	6 Apr	Head	DQ097079
9	22, M	Laranjal	Portel	No†	15 Mar	28 Mar	Feet	NA
10	2, M	Laranjal	Portel	No†	16 Mar	30 Mar	Head/feet	NA
11	58, M	Curupati	Viseu	Yes	30 Apr	17 May	NA	DQ097075
12	22, M	Curupati	Viseu	Yes	5 May	14 May	NA	NA

*NA, not available or unknown. †Diagnosis by histopathologic examination.

### Outbreak Areas

Many persons live in poor conditions in both municipalities where cases were reported. The municipality of Portel (50°57´W, 1°59´S), is situated in the Marajó Island region, state of Pará, and is ≈278 km distant from Belém, the state capital; access to Belém is by the Amazon River. Portel has ≈41,500 inhabitants (1.6 inhabitants/km^2^); ≈55% of them live in rural areas. The principal economic activities are cutting down timber, agriculture (especially cultivation of manioc), and hook-and-line fishing; cattle grazing is uncommon. In the Acuty-Perera River area, where cases were reported, the infected persons were from the following 3 communities: Ajará, Laranjal (Aparecida), and Tauaçu.

The municipality of Viseu, (50°49´W, 1°56´S) is in the Bragantina region of the state of Pará, 320 km distant from Belém, with access by highways. It has ≈52,893 inhabitants (10.2 inhabitants/km^2^); 68% live in rural areas. Rabies cases were reported in the Curupati community where ≈77 families lived. Economic activities include cultivation of manioc and hook-and-line fishing.

### Virus Isolation

All laboratory analyses were performed under pressurized containment cabinets, class II B2. From all patient specimens, homogenates were obtained as previously described (*14*). Briefly, 0.02 mL of each suspension in phosphate-buffered saline (pH 7.4) containing fraction V bovine albumin solution (0.75%), penicillin (100 IU/mL), and streptomycin (100 μg/mL), was injected intracerebrally into 12 newborn mice. After injection, the mice were observed daily for 3 weeks or until the animals became sick, when their brains were removed and used for immunofluorescence assay (IFA) or stored at –70°C for further molecular biology procedures.

### Detection and Characterization of Isolates

All human CNS samples and suckling mouse brains were used to prepare impression smears, which were examined by direct IFA with a fluorescent antirabies conjugate, as described elsewhere (*15*). All rabies virus strains isolated were antigenically typed by indirect IFA by using a panel of 8 monoclonal antibodies prepared against the viral nucleoprotein (Centers for Disease Control and Prevention, Atlanta, GA, USA) (*9**,**16*). Six original CNS samples (3066M, 3067M, 3068M, 3072M, and 3522M from Portel and 5214 from Viseu) were tested in a reverse transcription–PCR that amplifies a 1,352-bp fragment of the nucleoprotein gene with sense primer N1 5´-ATGGATGCCGACAAGATT 3´and anti-sense primer N2 5´-TTATGAGTCACTCGAATA 3´ as described by Carnieli et al. (*17*) by using Superscript II Reverse Transcriptase (Invitrogen, São Paulo, Brazil) and Taq DNA Polymerase (Invitrogen, Carlsbad, CA, USA), according to the manufacturer's instructions.

PCR products were excised from agarose gel, purified with QIAquick gel extraction kit (Qiagen, Valencia, CA, USA), and sequenced reaction with anti-sense primer and DYEnamic ET Dye Terminator (Amersham Biosciences, Piscataway, NJ, USA), according to the manufacturer's instructions, in 4 replicates. The sequences were resolved in a MegaBACE DNA sequencer (Amersham Biosciences).

**Figure 2:**
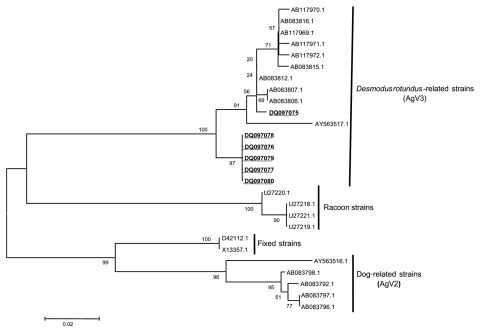
Neighbor-joining tree with K2P model based on partial nucleoprotein gene sequences of rabies virus from *Desmodus rotundus* AgV3, raccoons, fixed strains, and dog AgV2. Each taxon is represented by its respective GenBank accession number (human strains from the present study are in **bold** and underlined). Numbers at each node are 1,000 bootstrap replicate values; the bar indicates genetic distance.

The final sequence of each strain was aligned by the Clustal method with Bioedit (*18*) (http://www.mbio.ncsu.edu/BioEdit/bioedit.html) and MEGA 2.1 (http://www.megasoftware.net), with homologous sequences derived from GenBank (accession numbers shown in Figure 2). The alignment was used to build a neighbor-joining distance tree with the Kimura 2-parameter model and 1,000 bootstrap replicates with MEGA 2.1 (*19*).

## Results

Twenty-one cases of bat-transmitted rabies were reported (15 from Portel and 6 from Viseu). Twelve were confirmed by laboratory diagnostic procedures, and 9 cases (5 of Portel and 4 of Viseu) were confirmed by clinical and epidemiologic linkage. Of the laboratory-confirmed cases, 2 from Portel were diagnosed by histopathologic examination and 10 (8 from Portel and 2 from Viseu) were diagnosed by IFA or by virus isolation in suckling mice. Antigenic characterization showed variant 3 (AgV3), the primary reservoir of which is the vampire bat *Desmodus rotundus*. One additional rabies virus isolate obtained from a *D. rotundus* was also typed as AgV3 (E.S.T. da Rosa and P.F.C. Vasconcelos, unpub. data).

The nucleotide identity among the 6 sequences from the human rabies strains of this study was 99.3%; identity among the 5 strains from Portel was 100%, and identity among these and the strain from Viseu was 97.2%. These same 6 strains had an identity of 97% when compared to AgV3 *D. rotundus* strains and 82.8% when compared to AgV2 Brazilian dog strains. Furthermore, the nucleotide identity among the 6 human strains and strains related to raccoons was 85.2%; it was 84.5% when compared to fixed strains CVS and AV01 (GenBank accession nos. D42112.1 and X13357.1, respectively). The phylogenetic tree (Figure 2) shows a clustering pattern that is in accordance with each specific host or variant of the rabies virus, each cluster supported by a bootstrap value of at least 98%.

The 6 human isolates were genetically grouped in the *D. rotundus* cluster, supported by a bootstrap value as high as 1,000. The 5 strains from Portel grouped together in an exclusive polytomic subcluster, supported by a bootstrap value of 97%, while strain 5214M from Viseu grouped in a paraphyletic and more resolved subcluster among samples detected in *D*. *rotundus* and *Artibeus* spp. from Brazil and an AgV3 strain detected in a Brazilian cat (AY563517.1).

## Discussion

This is the first outbreak of vampire bat–transmitted rabies reported in Brazil in which rabies virus was isolated from humans and bats and in which the isolated strains were antigenically and genetically characterized. Previous reports of bat-transmitted outbreaks were based only on clinical and epidemiologic linkage, and in all of these outbreaks, infected persons were living in small, remote areas with difficult access in the Amazon region, including isolated Indian villages and clandestine gold mining areas in the states of central and northern Brazil. In these episodes, deaths were reported several weeks after the patients had died and therefore, no clinical specimens could be examined (*7**,**20*).

In contrast, although the outbreaks of Portel and Viseu occurred in isolated remote areas, access to them was facilitated by rivers (Portel) and highways (Viseu) and also by the fact that both areas were considerably closer to Belém, the capital of Pará State.

Moreover, in the last few years, improvement in the surveillance for rabies and several other less-studied infectious diseases by the Brazilian Ministry of Health and Secretary of Health for Pará State has resulted in a more sensitive system for detecting and investigating all relevant and unusual episodes suspected to be bat-transmitted rabies. The number of cases of bat-transmitted human rabies is, therefore, expected to increase: not only because of the improved epidemiologic surveillance system but also because of the reduction in opportunities for the bat's life cycle to be maintained in urban areas (*1**,**5**,**7**–**9**,**12*). An epidemiologic investigation of cases in humans in Portel and Viseu areas showed that vampire bat bites are common. Indeed, all victims had a history of bat bites, but bites were not considered a risk for acquiring rabies. Moreover, 1,558 persons in Portel (Acuty Perera River communities) reported >1 episode of vampire bat aggression, and many inhabitants reported several bat bites in the 12 months before the outbreak. All these persons received postexposure treatment (antirabies serum and 5 doses of diploid human cells vaccine). Persons reporting bat bites for >1 year (838 persons in the Acuty Perera River communities) received preexposure treatment (3 doses of diploid human cells vaccine). After treatment measures, cases of rabies by bat aggression were no longer reported. Moreover, in Portel County, 4,504 dogs and 1,789 cats were also vaccinated (*3*).

Nonetheless, the lack of clinicians to diagnose the first cases at local hospitals has contributed to the increased number of noninvestigated cases and for the delay in recognizing them as rabies. In Peru, the country with the highest prevalence of bat-transmitted rabies in the Americas, several epidemics have been recognized. Delay in recognizing the disease was associated with several outbreaks (*5**,**13*). In Portel and Viseu, all clinical cases were characterized as ascendant paralytic rabies. Patients exhibited paresis, paralysis, dyspnea and difficulties of speech, soreness or lethargy, photophobia, aerophobia, hydrophobia, and coma, symptoms similar to those previously reported in other vampire bat–transmitted rabies outbreaks (*1**,**5**,**7**,**21**–**24*). The topology of the neighbor-joining tree, shown in Figure 2, grouped all rabies strains according to each respective host and variant, which validates the sequenced region and the tree-building method; in addition, the nucleotide identity among AgV3 strains and the human strains studied here (97%) match the thresholds described for different variant-host associations (*25*). The distance-based phylogenetic analysis of the N gene, based on full-length (1,350 nt) or partial (200–300 nt) sequencing, allows highly statistically supported clusterings for each rabies virus variant or each host-specific variant; the method is the most efficient in rabies molecular epidemiology (*26*).

These results lead to the conclusion that all strains of rabies virus isolated from humans during the rabies outbreak in northern Brazil in 2004 are related to the *D. rotundus* variant commonly found in Brazil, which supports the data generated by antigenic typing. Nevertheless, since all 5 sequenced strains from Portel grouped in different subclusters when compared with the sequenced strain from Viseu, this finding might be a sign that regional patterns of lineages of AgV3 rabies virus exist. Because the 2 municipalities are 530 km apart and in different ecologic and geographic regions, the Portel subclusters represent a unique and exclusive lineage. Whether the strain involved in the Viseu outbreak is in fact, monophyletic with all *D. rotundu*s strains used in the phylogenetic analysis or could give rise to other paraphyletic subclusters still remains to be answered by analysis of more samples from the same area.

The complete identity among the 5 Portel strains might be due to the high attack rates reported in this specific outbreak, because the same virus lineage or subclusters would be rapidly transmitted by a homogeneously infected population of vampire bats sharing an exclusive lineage of rabies virus. Subclusters in AgV3 from *D. rotundus* are not an uncommon binding, and strains from the same area are likely to cluster together (*27*). In Latin America, area-specific clusterings have been described for AgV3 in Argentina, Venezuela, and Mexico, where clusterings divergent from those in Brazil are known to occur (*6**,**9**,**12**,**28*).

The genetic data obtained from the human strains studied here might be used to follow in a more accurate way, the population of *D. rotundus* involved in transmission when the rabies virus strains detected in these bats and regional ecologic information about these become available. This information might provide a powerful tool to help understand the factors that facilitated the outbreak and prevent others in the future.

The isolation of a strain from the vampire bat *D. rotundus* in Breves, a municipality bordering Portel (Figure 1) and close to where human infections were reported, is definitively incriminates this species in the transmission of rabies virus in Pará State (E.S.T. da Rosa and P.F.C. Vasconcelos, unpub. data).

**Figure 3:**
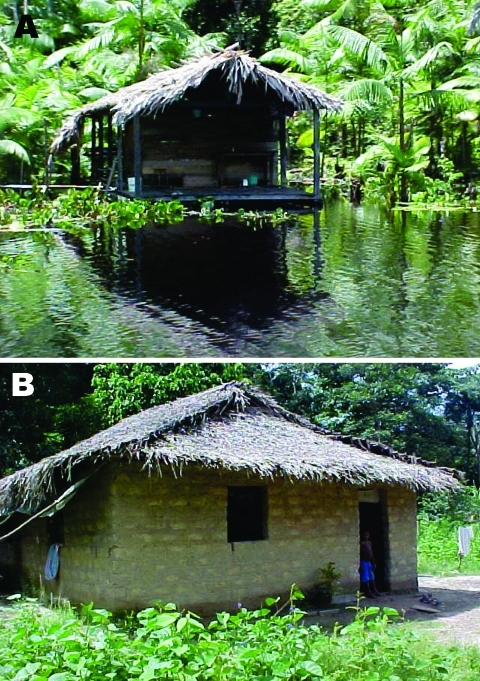
Typical human dwellings in the Acuty Perera River region, Portel (A), and in the Curupati area, Viseu (B). Note in (A) a house without walls on the Acuty Perera River.

Previous reports showed a rabid infection frequency ranging from 0% to 3% for *D. rotundus*, which is associated with high or low endemicity (*29*). In the outbreaks described in this article, rabies virus was only isolated near the Portel area (Breves municipality) from a single *D. rotundus* among 23 of 132 bats studied. The aggression of vampire bats in these remote areas may be because persons live in unprotected dwellings (houses either without walls or without windows and doors) as shown in Figure 3, and the number of wild animals, cattle or equines, is small. Moreover, in June 2005, 9 other vampire bat–transmitted rabies cases in humans were reported in communities of Augusto Correa Municipality (Figure 1), which borders Viseu (E.S.T. da Rosa and P.F.C. Vasconcelos, unpub. data).

Finally, public health campaigns should carried out to alert inhabitants of remote, small communities in the Amazon region to the risk of bat bites in transmitting rabies, and the need for all persons who report attacks of bats to undergo postexposure treatment to prevent other cases of vampire bat–transmitted rabies. Also, ecologic studies should be initiated to clarify the dynamics of rabies infection between populations of *D. rotundus* in affected areas.

## References

[1] Rupprecht CE, Hanlon CA, Hamachudha T. Rabies re-examined. Lancet Infect Dis. 2002;2:327–43. 10.1016/S1473-3099(02)00287-6 12144896

[2] Warrell MJ, Warrell DA. Rabies and other lyssavirus diseases. Lancet. 2004;363:959–69. 10.1016/S0140-6736(04)15792-9 15043965

[3] Ministério da Saúde. Surto de raiva human transmitida por morcegos no município de Portel-Pará, Março/Abril de 2004. Boletim Eletrônico Epidemiológico (Brasília). 2004;4:2–5.

[4] Diaz AM, Papo S, Rodriguez A, Smith JS. Antigenic analysis of rabies-virus isolates from Latin America and the Caribbean. Zentralbl Veterinarmed B. 1994;41:153–60. 7801717 10.1111/j.1439-0450.1994.tb00219.x

[5] Warner CK, Zaki SR, Shieh W-J, Whitfield SG, Smith JS, Orciari LA, Laboratory investigation of human deaths from vampire bat rabies in Peru. Am J Trop Med Hyg. 1999;60:502–7. 10466985 10.4269/ajtmh.1999.60.502

[6] Velasco-Villa A, Gomez-Sierra M, Hernandez-Rodriguez G, Juarez-Islas V, Melendez-Felix A, Vargas-Pino F, Antigenic diversity and distribution of rabies virus in Mexico. J Clin Microbiol. 2002;40:951–8. 10.1128/JCM.40.3.951-958.2002 11880422 PMC120240

[7] Schneider MC, Santos-Burgoa C, Aron J, Munoz B, Ruiz-Velazco S, Uieda W. Potential force of infection of human rabies transmitted by vampire bats in the Amazonian region of Brazil. Am J Trop Med Hyg. 1996;55:680–4. 9025698 10.4269/ajtmh.1996.55.680

[8] Schneider MC, Aron J, Santos-Burgoa C, Uieda W, Ruiz-Velazco S. Common vampire bat attacks on humans in a village of the Amazon region of Brazil. Cad Saude Publica. 2001;17:1531–6. 10.1590/S0102-311X2001000600038 11784915

[9] De Mattos CC, De Mattos CA, Loza-Rubio E, Aguilar-Setién A, Orciari LA, Smith JS. Molecular characterization of rabies virus isolates from Mexico: implications for transmission dynamics and human risk. Am J Trop Med Hyg. 1999;61:587–97. 10548293 10.4269/ajtmh.1999.61.587

[10] Rohde RE, Mayes BC, Smith JS, Neill SU. Bat rabies, Texas, 1996–2000. Emerg Infect Dis. 2004;10:948–52. 15200840 10.3201/eid1005.030719 PMC3323228

[11] Morimoto K, Patel M, Corisdeo S. Hooper Dc, Fu ZF, Rupprecht CE. Characterization of a unique variant of bat rabies virus responsible for newly emerging human cases in North America. Proc Natl Acad Sci U S A. 1996;93:5653–8. 10.1073/pnas.93.11.5653 8643632 PMC39303

[12] de Mattos CA, de Mattos CC, Smith JS, Miller ET, Papo S, Utrera A, Genetic characterization of rabies field isolates from Venezuela. J Clin Microbiol. 1996;34:1553–8. 8735118 10.1128/jcm.34.6.1553-1558.1996 PMC229062

[13] Lopez A, Mirand P, Tejada E, Fishbein DB. Outbreak of human rabies in the Peruvian jungle. Lancet. 1992;339:408–11. 10.1016/0140-6736(92)90088-K 1346669

[14] Koprowski H. The mouse inoculation test. In: Meslin FX, Kaplan MM, Koprowski H, editors. Laboratory techniques in rabies. Geneva: World Health Organization; 1996. p. 80–7.

[15] Dean DJ, Abelseth MK. The fluorescent antibody test. In: Kaplan MM, Koprowski E, editors. Laboratory techniques in rabies, 3rd edition. Geneva: World Health Organization; 1973. p. 73–84. 4219510

[16] Favoretto SR, Carrieri ML, Cunha EM, Aquiar EA, Silva LH, Sodre MM, Antigenic typing of Brazilian rabies virus samples isolated from animals and humans, 1989-2000. Rev Inst Med Trop Sao Paulo. 2002;44:91–5. 10.1590/S0036-46652002000200007 12048546

[17] Carnieli Jr. Brandão PE, Macedo CI, Castilho JG, Zanetti CR, Carrieri ML, et al. Rabies in cats caused by rabies virus variants of bats: a new challenge to public health. In: The XV International Conference of Rabies in the Americas, 2004, Santo Domingo. Santo Domingo, Dominican Republic: Pan American Health Organization; 2004. pp. 37–8.

[18] Hall TA. BioEdit: a user-friendly biological sequence alignment editor and analysis program for Windows 95/98/NT. Nucl Acids Symp Ser. 1999;41:95–8.

[19] Kumar S, Tamura K, Jakobsen IB, Nei M. MEGA2: Molecular evolutionary genetics analysis software. Tempe (AZ): Arizona State University; 2001. 10.1093/bioinformatics/17.12.1244 11751241

[20] Mayen F. Haematophagous bats in Brazil, their role in rabies transmission, impact on public health, livestock industry and alternatives to an indiscriminate reduction of bat population. J Vet Med. 2003;B50:469–72. 10.1046/j.1439-0450.2003.00713.x 14720182

[21] Hurst EW, Pawan JL. An outbreak of rabies in Trinidad without history of bites and with symptoms of acute ascending paralysis. Lancet. 1931;ii:622–5. 10.1016/S0140-6736(01)07332-9 14405537

[22] Hurst EW, Pawan JL. A further account of the Trinidad outbreak of acute rabies myelitis. J Pathol Bacteriol. 1932;35:301–21. 10.1002/path.1700350302

[23] Pawan JL. Rabies in the vampire bat of Trinidad, with special reference to the clinical course and the latency of infection. Ann Trop Med Parasitol. 1936;30:101–29. 14431118

[24] Nehaul BBG. Rabies transmitted by bats in British Guiana. Am J Trop Med Hyg. 1955;4:550–3. 14376780 10.4269/ajtmh.1955.4.550

[25] Smith JS, Orciari LA, Yager PA, Seidel HD, Warner CK. Medline Epidemiologic and historical relationships among 87 rabies virus isolates as determined by limited sequence analysis. J Infect Dis. 1992;166:296–307. 10.1093/infdis/166.2.296 1634801

[26] Smith JS. Molecular epidemiology. In: Jackson AC, Wunner WH, editors. Rabies. San Diego: Academic Press; 2002. p. 79–111.

[27] Ito M, Arai YT, Itou T, Sakai T, Ito FH, Takasaki T, Genetic characterization and geographic distribution of rabies virus isolates in Brazil: identification of two reservoirs, dogs and vampire bats. Virology. 2001;284:214–22. 10.1006/viro.2000.0916 11384221

[28] Cisterna D, Bonaventura R, Caillou S, Pozo O, Andreau ML, Fontana LD, Antigenic and molecular characterization of rabies virus in Argentina. Virus Res. 2005;109:139–47. 10.1016/j.virusres.2004.10.013 15763144

[29] Côrtes VA, Souza LC, Uieda W, Figueiredo AC. Abrigos diurnos e infecção rábica em morcegos de Botucatu, São Paulo, Brasil. Vet e Zoot São Paulo. 1994;6:179–86.

